# A meta-analysis of the influence of traditional Chinese exercises on cognitive function in the elderly

**DOI:** 10.3389/fpsyg.2025.1516197

**Published:** 2025-04-08

**Authors:** Xueqi Liu, Dewei Sun, Weiran Zhao, Xiaoying Zhang, Yixin Wu

**Affiliations:** College of Exercise and Health, Shenyang Sport University, Shenyang City, China

**Keywords:** traditional Chinese exercises, elderly, cognitive function, cognitive impairment, meta-analysis

## Abstract

**Background:**

This study seeks to assess the impact of traditional Chinese exercises (Tai Chi, Baduanjin, Yijinjing, Wuqinxi, and Liuzijue) on cognitive function in older adults through a systematic review and meta-analysis. It examines their effects on global cognitive performance, as well as specific cognitive domains, providing robust evidence to support the enhancement of cognitive function in the elderly.

**Methods:**

A thorough search was executed across eight key databases, including PubMed, Embase, Cochrane Library, EBSCO, Web of Science, Wanfang Data, the Chinese Science and Technology Journal Database, and China National Knowledge Infrastructure. The quality of the studies that met the inclusion criteria was assessed using the Cochrane Collaboration tool for evaluating risk of bias. Data synthesis was performed using Review Manager 5.4, where pooled intervention outcomes were expressed as mean differences (MD) alongside their 95% confidence intervals (CI). Additional sub-group analysis was conducted to explore potential factors contributing to heterogeneity.

**Results:**

This systematic review and meta-analysis evaluated data from 29 randomized controlled trials, encompassing a total of 2,489 participants. The results demonstrate that traditional Chinese exercises substantially improve language abilities (Category Verbal Fluency: MD = 0.90, 95% CI: 0.38 to 1.41), executive function (TMT B: MD = −13.70, 95% CI: −16.06 to −11.35), short-term memory (MD = 0.85, 95% CI: 0.42 to 1.29), and long-term delayed recall (MD = 1.39, 95% CI: 0.72 to 2.06). Sub-group analysis indicated that baseline cognitive function plays a critical role in determining the effectiveness of the intervention. Patients with cognitive impairment derive significantly greater benefits from traditional Chinese exercise interventions than those with normal cognitive function.

**Conclusion:**

This study found that the traditional Chinese exercises can enhance cognitive function in the elderly, particularly in those with mild cognitive impairment. The effects of traditional Chinese exercises differed across various cognitive domains, indicating that when utilizing traditional Chinese exercise as an intervention, it is crucial to consider the specific cognitive status of the patient to design precisely tailored intervention strategies.

**Systematic review registration:**

https://www.crd.york.ac.uk/PROSPERO/display_record.php?ID=CRD42024535287, identifier [CRD42024535287].

## 1 Introduction

As individuals age, they encounter the challenge of gradual cognitive decline, with some progressing to cognitive impairment (Cheng A. et al., 2022). This condition, characterized by difficulties in memory, language, attention, and executive function, arises from the degeneration or damage of brain functions (Wang et al., 2021). Cognitive impairment can significantly diminish the independence and quality of life of older adults, while also increasing the risk of premature mortality (Park et al., 2023).

Research has established a significant positive correlation between physical exercise and cognitive function (Chen et al., 2024; Huang et al., 2022; Ludyga et al., 2023). Exercise plays a vital role in preventing and alleviating cognitive decline in older adults by promoting the production of neurotrophic factors and facilitating neuronal repair (Wayne et al., 2014). Yet the effects of various physical activities on cognitive function and brain health differ. Huang et al. (2022) observed that multicomponent exercise (A combination of 2 exercises) has a pronounced effect in preventing overall cognitive decline in patients with mild cognitive impairment (MCI). Conversely, Li H. et al. (2022) found that moderate-to-high intensity aerobic and resistance exercises offer limited cognitive benefits and may pose a risk of adverse events. Therefore, it is particularly important to design exercise programs for older adults that are of moderate intensity, highly safe, and demonstrably effective.

Traditional Chinese exercises (TCEs) are deeply embedded in China’s cultural heritage, characterized by gentle, slow movements that emphasise the coordination of breath and movement, while fostering the harmonious integration of body and mind (Chen et al., 2019; Guo et al., 2024). These practices form a comprehensive system that unites physical movement, breathing techniques, and mental regulation, with the aim of enhancing physical health and promoting overall well-being (Gao et al., 2021). TCE practices, including Tai Chi, Baduanjin, Yijinjing, Wuqinxi, and Liuzi Jue, are particularly suitable for individuals of all ages, owing to their moderate difficulty and intensity, along with their well-documented health benefits (Zhang et al., 2020). Supported by the proactive promotion of the General Administration of Sport of China, these practices have gained widespread popularity, particularly among the elderly, and have become a widely adopted choice for daily fitness routines (Yang et al., 2022).

Research indicates that TCEs can significantly enhance physical function in older adults and effectively prevent and manage chronic diseases (Cheng M. et al., 2022; Klarod et al., 2024; Li et al., 2020). The distinctive combination of breath control, slow and continuous movements, and psychological adjustment in TCEs promotes improved blood circulation, enhanced organ function, muscle and tendon activation, and optimized breathing patterns (Zhou et al., 2022). These physiological and psychological benefits collectively contribute to brain health, offering a basis for the prevention and mitigation of cognitive decline in the elderly. The long-term practice of TCEs not only alleviates stress, regulates mood, and fosters mind–body harmony, but also holds the potential to enhance cognitive function through mechanisms such as increased neural plasticity and improved cerebral blood flow (Ren et al., 2021; Tsai et al., 2018; Yang et al., 2022).

Although existing research has underscored the benefits of TCEs for cognitive function, it is constrained by small sample sizes, methodological inconsistencies, and variations in assessment tools, resulting in conflicting findings (Yao et al., 2023; Zhou et al., 2022). Current meta-analyses predominantly focus on elderly individuals with MCI, largely overlooking the effects on cognitively healthy individuals and those with other forms of cognitive dysfunction. However, TCEs may not only enhance cognitive function in individuals with pre-existing cognitive impairments but may also contribute to the prevention of cognitive decline in those who are cognitively healthy. Consequently, a comprehensive evaluation of TCE effects across different cognitive states in older populations is crucial for a more nuanced understanding of its potential efficacy. To address this gap, the present study utilises standardised assessment tools and stringent inclusion criteria to systematically evaluate the intervention effects across a range of cognitive status groups, overcoming the limitations of prior studies and offering more robust evidence to inform clinical practice.

## 2 Methods

This meta-analysis was conducted following the guidelines outlined by the Preferred Reporting Items for Systematic Reviews and Meta-Analyses (PRISMA) (Page et al., 2021).

### 2.1 Registration

This study has been registered in the Prospective Register of Systematic Reviews (CRD42024535287).

### 2.2 Literature source

We conducted searches in five English databases (PubMed, Embase, Cochrane Library, EBSCO, and Web of Science) and three Chinese databases (Wanfang Data, Chinese Science and Technology Journal Database, and China National Knowledge Infrastructure). The search spanned from the inception of each database until 1 May 2024, with no language restrictions. The search terms included Tai Chi, Baduanjin, Yijinjing, Wuqinxi, Liuzijue, cognition, cognitive function, executive control, memory, elderly, older adults, randomized controlled trial, controlled clinical trial, and randomized clinical trial. Corresponding Chinese terms were used for searches in the Chinese databases. The detailed search strategy is provided in Supplementary Table S1. We also searched for gray literature using OpenGrey, Mednar, and the World Health Organization’s search portal, and screened the reference lists of the included studies to identify additional articles that could be eligible. The detailed search strategy is provided in Supplementary Table S2.

### 2.3 Inclusion and exclusion criteria

The inclusion and exclusion criteria were established using the PICOS framework (Li et al., 2023).

#### 2.3.1 Inclusion criteria

- Participants: The study population included those with an average age of 60 years and above (Guo et al., 2024) and who have either normal cognitive function, mild cognitive impairment, or experience other forms of cognitive dysfunction, such as cognitive decline, risk of cognitive deterioration, or self-reported cognitive decline.
- Intervention: The experimental group exclusively received interventions involving traditional Chinese physical exercises, including Tai Chi, Baduanjin, Yijinjing, Wuqinxi, and Liuzijue.
- Control Group: The control group received either standard care, no intervention, health education, or other types of exercise interventions.
- Outcomes: At least one outcome measure assessed cognitive function.
- Study Design: Randomized controlled trials.
- Publication Type: Journal articles.

#### 2.3.2 Exclusion criteria

- Studies involving the same cohort of patients at different time points.
- Studies with incomplete or irretrievable original data, or those containing evident errors.
- Non-clinical trial research, such as reviews, basic research, dissertations, case reports, and conference abstracts.

### 2.4 Study selection and data extraction

Two researchers independently screened the studies based on the predefined inclusion and exclusion criteria. Any discrepancies were resolved through discussion with the corresponding author to reach a consensus. The following data were extracted from each study: publication details (authors, year of publication), basic characteristics (sample size, type of intervention), participant characteristics (age, sample size, cognitive status), and study design (randomized controlled trial). Pre- and post-intervention outcome data (mean ± standard deviation) were extracted to assess the impact of the TCEs on cognitive function. For studies reporting data at multiple time points, only the results measured immediately after the intervention were included. When articles provided only standard error (SE), confidence intervals (CI), or interquartile range (IQR), these were converted to mean and standard deviation using the RevMan 5.4 calculator.

### 2.5 Quality assessment

Two independent reviewers evaluated the risk of bias in the studies included in this analysis using the Risk of Bias 2 (RoB 2) tool, a revised version of the Cochrane Risk of Bias tool designed specifically for randomised trials. The assessment encompassed the following domains: randomisation process, deviations from intended interventions, missing outcome data, measurement of the outcome, and selection of the reported results (Sterne et al., 2019). Bias risk was categorised into three levels: low risk, some concerns, and high risk. Any discrepancies between reviewers were resolved through discussion and by consulting the corresponding author until a consensus was reached.

### 2.6 Statistical analysis

This study employed RevMan 5.4 to conduct a meta-analysis using a frequentist approach to evaluate the impact of TCEs on cognitive function in older adults. Continuous variables were reported as mean differences (MD) with 95% confidence intervals (CI), and with MD employed to account for differences in measurement units. Heterogeneity was assessed using the Q test and I^2^ statistic (Hosseini Hosseini Koukamari et al., 2024). A fixed-effect model was applied if *I*^2^ ≤ 50% or *p* ≥ 0.1, indicating low heterogeneity. If *I*^2^ > 50% or *p* < 0.1, indicating high heterogeneity, a random-effects model was used. Sensitivity analysis was performed by sequentially excluding each study and recalculating heterogeneity. If excluding a study led to a significant change in heterogeneity, this suggested that the study had a substantial impact on the results. If no significant change was observed, the results were considered robust and not influenced by any single study. Publication bias was evaluated using a funnel plot, with the significance level set at α = 0.05.

## 3 Results

### 3.1 Search results

**Figure 1:**
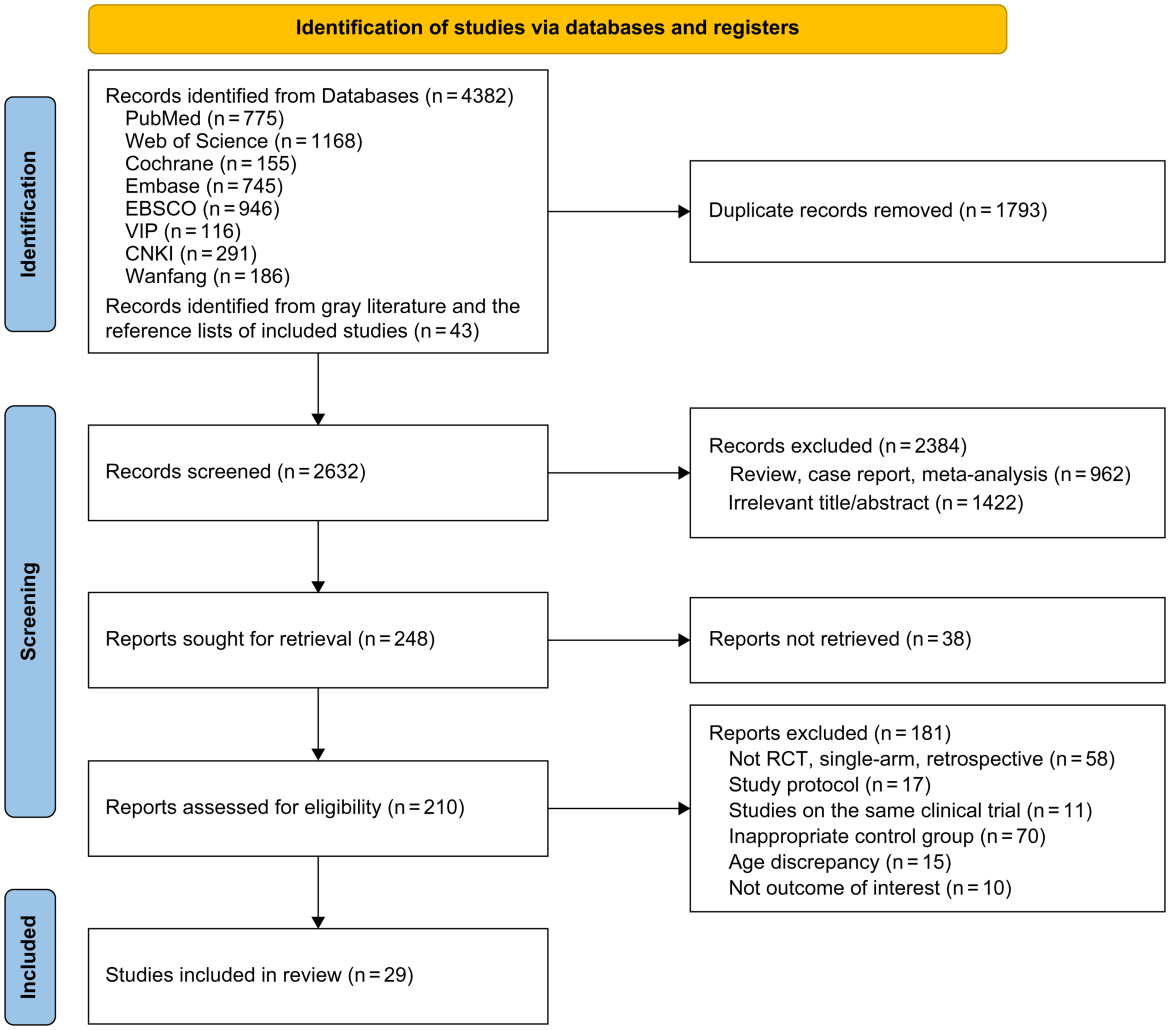
Study selection process.

A total of 4,425 studies were retrieved. After screening the titles and abstracts, we excluded 2,384 irrelevant studies and 1,793 duplicates. We then assessed the full texts of the remaining 248 studies. Of these, 219 were excluded for the following reasons: full text not available (n = 38), non-randomized controlled trials (n = 58), study protocols (n = 17), duplicate studies arising from the same clinical trial (n = 11), inappropriate control groups (n = 70), age criteria not met (n = 15), and irrelevant outcomes (n = 10). Ultimately, 29 studies were included in the analysis. The study selection process is depicted in Figure 1.

### 3.2 Characteristics of included studies

Table 1 summarizes the 29 studies included in this analysis (Cheng et al., 2014; Chen et al., 2023; Chi et al., 2017; Haiming, 2012; JianPing et al., 2016; Jinyan et al., 2018; Lam et al., 2014; Lin et al., 2023; Liu et al., 2022; Li F. et al., 2022; Mortimer et al., 2012; Na-na and Chao, 2019; Nguyen and Kruse, 2012; Qiu, 2016, 2017; Sungkarat et al., 2018; Sun et al., 2015; Su et al., 2021; Tao et al., 2015; Tao et al., 2018; Tao et al., 2019; Taylor-Piliae et al., 2010; Tsai et al., 2013; Wang X. et al., 2024; Wan et al., 2022; Wenli et al., 2023; Xia et al., 2023; Yu et al., 2022; Zheng et al., 2021) published between 2010 and 2023. Sample sizes ranged from 22 to 353 participants per study, with all studies including both male and female participants. The participants comprised older adults with normal cognitive function (K = 6), those with MCI (K = 15), and individuals with other types of cognitive dysfunction, including at-risk cognitive decline, amnestic cognitive impairment, and self-reported memory issues (K = 8).

**Table 1:** Characteristics of the included studies.

Author (year)	Study design	Country/Region	Study participants	Cognitive function status	Sample size N and gender (M/F)	Intervention (frequency)	Control	Relevant outcome (measurements)
Chen et al. (2023)	RCT	China	Older people (mean age: EX:67.56 ± 4.99 CO:67.62 ± 5.35)	MCI	N:218 EX:49/58 CO:51/60	Tai Chi (24 weeks, 60 min/session, 3 times per week)	NO	(1)，(9), (11)
Cheng et al. (2014)	RCT	China	Older people (mean age: EX:81.80 ± 7.40 CO:80.90 ± 7.20)	MCI or CDR 0.5	N:74 EX:12/23 CO:14/25	Tai Chi (12 weeks, 60 min/session, 3 times per week)	Simple handicrafts	(2)
Chi et al. (2017)	RCT	China	Older people (mean age: EX:66.20 ± 5.10 CO:64.70 ± 6.50)	MCI	N:60 EX:12/18 CO:14/16	Liuzijue (12 weeks, 90 min/session, 1 time per week)	NO	(2)
Li F. et al. (2022)	RCT	America	Older people (mean age: EX:74.50 ± 5.60 CO:74.90 ± 6.30)	MCI	N:46 EX:14/8 CO:9/15	Tai Chi (16 weeks, 60 min/session, 2 times per week)	Stretching exercises	(1), (6), (7), (9), (11)
Haiming (2012)	RCT	China	Older people (mean age: EX:68.17 ± 3.50) CO:67.9 ± 5.00	Normal cognitive function	N:40 EX:10/12 CO:9/9	Wuqinxi (24 weeks, 60 min/session, 6 times per week)	NO	(1), (2)，(11)
JianPing et al. (2016)	RCT	China	Older people (mean age: EX:67.00 ± 10.40 CO:69.00 ± 10.70)	MCI	N:70 EX:21/14 CO:19/16	Badaunjin (24 weeks, 60 min/session, 6 times per week)	NO	(1)
Jinyan et al. (2018)	RCT	China	Older people (mean age: EX:63.50 ± 3.60 CO: 62.60 ± 2.90)	Normal cognitive function	N:73 EX:35 CO:38	Yijinjing, (Learning stage: 2 weeks, 60 ~ 90 min/session, ≥5 times per week; Practice stage: 8 weeks,60 ~ 90 min/session, ≥5 times per week)	NO	(1)
Lam et al. (2014)	RCT	China	Older people (mean age: EX:77.20 ± 6.30 CO:78.30 ± 6.60)	CDR 0.5 or a-MCI	N:389 EX:46/125 CO:46/172	Tai Chi (48 weeks, 30 min/session, 3 times per week)	Stretching and relaxation exercises	(2), (4), (6), (7), (8), (9), (11)
Lin et al. (2023)	RCT	China	Older people (mean age: EX:67.68 ± 5.19 CO:65.35 ± 5.15)	MCI	N:102 EX:19/32 CO:20/31	Tai Chi (24 weeks, 60 min/session, 3 times per week)	Health education	(1)
Liu et al. (2022)	RCT	China	Older people (mean age: EX:73.20 ± 6.30 CO:73.40 ± 6.50)	MCI	N:34 EX:5/12 CO:6/11	Tai Chi (12 weeks, 50 min/session, 3 times per week)	NO	(1), (8), (9)
Mortimer et al. (2012)	RCT	China	Older people (mean age: EX:67.30 ± 5.30 CO:68.20 ± 6.50)	Normal cognitive function	N:60 EX:11/19 CO:9/21	Tai Chi (40 weeks, 50 min/session, 3 times per week)	NO	(6), (7), (8), (9), (11)
Na-na and Chao (2019)	RCT	China	Older people (mean age: EX:68.22 ± 9.84 CO:65.62 ± 9.34)	a-MCI	N:62 EX:14/17 CO:15/16	Tai Chi (24 weeks, 60 min/session, 3 days per week)	Health education	(1), (2)
Nguyen and Kruse (2012)	RCT	Vietnam	Older people (mean age: EX:69.23 ± 5.30 CO:68.73 ± 4.95)	Normal cognitive function	N:96 EX:24/24 CO:24/24	Tai Chi (24 weeks, 60 min/session, 2 times per week)	NO	(8), (12), (9)
Qiu (2016)	RCT	China	Older people (mean age: 67.13 ± 10.39)	MI	N:98 41/57	Baduanjin (24 weeks, 60 min/session)	Health education	(1), (2)
Qiu (2017)	RCT	China	Older people C (mean age: EX:68.74 ± 4.64 CO:69.23 ± 4.82)	MCI	N:98 EX:28/19 CO:30/17	Baduanjin (24 weeks, 1 time/session, 6 times/week)	Routine care	(1), (2)
Su et al. (2021)	RCT	China	Older people (mean age: EX:64.40 ± 6.57 CO:65.37 ± 6.31)	Subjective memory complaints	N:70 EX:16/19 CO:18/17	Baduanjin (12 weeks, 60 min/session, 5 times per week)	Gymnastics practice	(3), (4), (5), (8), (9)
Sun et al. (2015)	RCT	China	Older people (mean age: EX:68.30 ± 5.90 CO:70.10 ± 5.70)	Normal cognitive function	N:138 EX:14/58 CO:20/46	Tai Chi (24 weeks, 60 min/session, 2 times per week)	Other activities	(2)
Sungkarat et al. (2018)	RCT	Thailand	Older people (mean age: EX:68.30 ± 6.70 CO:67.50 ± 7.30)	a-MCI	N:66 EX:2/31 CO:7/26	Tai Chi (24 weeks, 50 min/session, 3 times per week)	Health education	(10)
Tao et al. (2015)	RCT	China	Older people (mean age: 69.00 ± 10.30)	MCI	N:60 38/22	Baduanjin (24 weeks, 60 min/session, 6 times per week)	NO	(1)
Tao et al. (2018)	RCT	China	Older people (mean age: EX:71.23 ± 5.53 CO:71.60 ± 5.29)	MCI	N:60 EX:21/9 CO:17/13	Baduanjin, (Learning stage: 2 weeks, 45 min/session, 3 times per week; Practice stage: 24 weeks, 60 min/session, 6 times per week)	NO	(1)
Tao et al. (2019)	RCT	China	Older people (mean age: EX:66.17 ± 4.17 CO:65.97 ± 5.66)	MCI	N:40 EX:5/15 CO:6/14	Baduanjin (24 weeks, 60 min/session, 3 times per week)	NO	(1)
Taylor-Piliae et al. (2010)	RCT	America	Older people (mean age: EX:70.60 ± 5.90 CO:68.20 ± 6.20)	Normal cognitive function	N:93 EX:13/24 CO:16/40	Tai Chi (24 weeks, 60 min/session, 5 times per week [2 sessions/class training and 3 sessions/home training])	Attention control	(6), (7), (11)
Tsai et al. (2013)	RCT	America	Older people (mean age: EX:78.89 ± 6.91 CO:78.93 ± 8.30)	MCI	N:55 EX:6/22 CO:9/18	Tai Chi (20 weeks, 20–40 min/session, 3 times per week)	Attention control	(2)
Wan et al. (2022)	RCT	China	Older people (mean age: EX:67.31 ± 5.58 CO:64.71 ± 5.07)	cognitive frailty with MCI	N:50 EX:9/17 CO:12/12	Baduanjin (24 weeks, 60 min/session, 3 times per week)	Health education	(1)
Wang X. et al. (2024)	RCT	China	Older people (mean age: EX:66.90 ± 4.54 CO:67.64 ± 5.49)	cognitive frailty	N:120 EX:11/49 CO:14/46	Baduanjin (24 weeks, 60 min/session, 3 times per week)	NO	(1), (8), (9), (11)
Wenli et al. (2023)	RCT	China	Older people (mean age: EX: 67.50 ± 7.30 CO: 68.60 ± 7.50)	a-MCI	N:63 EX:9/22 CO:10/22	Tai Chi (12 weeks, 50 min/session, 3 times per week)	Health education	(1), (4), (5)
Xia et al. (2023)	RCT	China	Older people (mean age: EX:66.16 ± 4.16 CO:65.41 ± 4.90)	MCI	N:90 EX:14/31 CO:10/35	Baduanjin (24 weeks, 60 min/session, 3 times per week)	NO	(1), (3), (4), (5), (10)
Yu et al. (2022)	RCT	China	Older people (mean age: EX:67.30 ± 4.20 CO:67.60 ± 8.10)	MCI	N:22 EX:3/7 CO:2/10	Tai Chi (24 weeks, 60 min/session, 3 times per week)	NO	(5), (6), (7), (8), (9), (10), (11)
Zheng et al. (2021)	RCT	China	Older people (mean age: EX:65.79 ± 4.35 CO:65.86 ± 5.28)	MCI	N:46 EX:6/17 CO:6/17	Baduanjin (24 weeks, 60 min/session, 3 times per week)	NO	(1)

RCT, randomized controlled trial; N, number; M, male; F, female; EX, exercise Group; CO, control group; CDR, clinical dementia rating; OA, knee osteoarthritis; (1) MoCA; (2) MMSE; (3) Immediate recall; (4) Short-term delayed recall; (5) Long delayed recall; (6) Digit span forward; (7) Digit span backward; (8) TMT A; (9) TMT B; (10) Part B-A difference score; (11) Category verbal fluency.

### 3.3 Quality of the included studies

**Figure 2:**
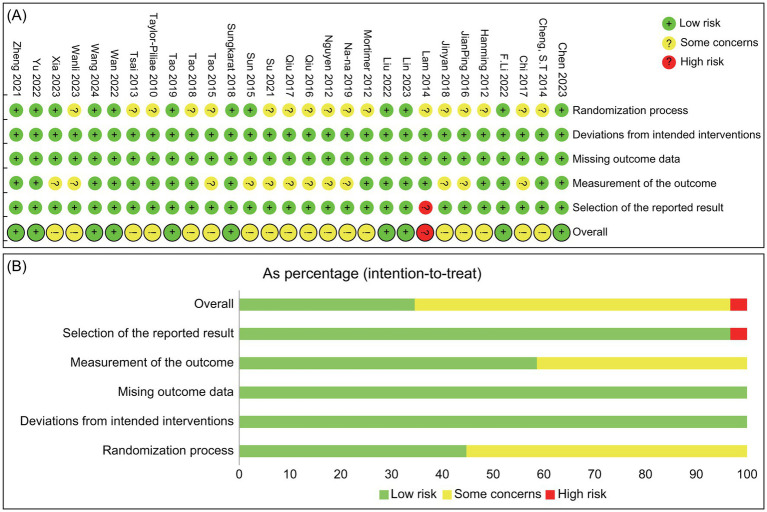
Risk of bias assessment for included studies: **(A)** Risk of bias summary; **(B)** Risk of bias graph.

Of the 29 studies included in this analysis, 13 were rated as low risk in the randomization process domain, while 16 raised some concerns, primarily owing to the unclear concealment of allocation. All studies were classified as low risk for both deviations from intended interventions and missing outcome data. In the measurement of the outcome domain, 17 studies were rated as low risk, with 12 raising some concerns. Regarding the selection of reported results, 28 studies were deemed low risk, and 1 study was rated as high risk. Overall, most studies exhibited a low risk of bias, though some concerns were noted in specific areas, particularly with respect to randomization and outcome measurement. Further specifics are depicted in Figure 2.

### 3.4 Outcomes

#### 3.4.1 Primary outcomes: overall cognitive function

**Figure 3:**
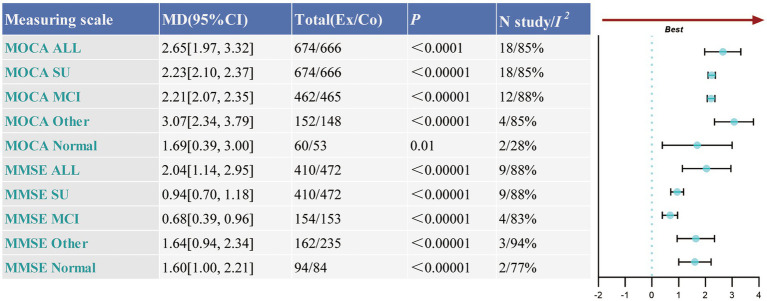
Comprehensive forest plots of overall cognitive function. Ex, experimental group; Co, control group; All, meta-analysis results encompassing all patients; SU, results following sub-group analysis; MCI, mild cognitive impairment; Normal, older adults with normal cognitive function; Other, other types of cognitive impairments.

Eighteen studies involving 1,340 participants used the Montreal Cognitive Assessment (MoCA) scale, while nine studies involving 882 participants employed the Mini-Mental State Examination (MMSE) scale to assess global cognitive function. Both sets of results demonstrated high heterogeneity (MoCA: *p* < 0.00001, *I*^2^ = 85%; MMSE: *p* < 0.00001, *I*^2^ = 88%). Analysis using a random-effects model revealed that TCEs were significantly more effective than the control group in improving global cognitive function (MoCA: MD = 2.65, 95% CI: 1.97–3.32, *p* < 0.00001; MMSE: MD = 2.04, 95% CI: 1.14–2.95, *p* < 0.00001). Sensitivity analysis confirmed the robustness of these findings. Sub-group analysis revealed that TCE interventions provided benefits across various cognitive function statuses in elderly individuals, although the magnitude of these effects was influenced by the assessment scale employed. For elderly individuals with other forms of cognitive dysfunction, significant cognitive improvements were observed post-intervention on both the MoCA (MD = 3.07, 95% CI: 2.34–3.79, *p* < 0.00001) and MMSE (MD = 1.64, 95% CI: 0.94–2.34, *p* < 0.0001) scales, with the most pronounced improvements in this group. In individuals with MCI, improvements were noted on both scales; however, the MoCA scale showed a moderate effect compared to the other two groups (MD = 2.21, 95% CI: 2.07–2.35, *p* < 0.0001), whereas the MMSE scale demonstrated the smallest improvement (MD = 0.68, 95% CI: 0.39–0.96, *p* < 0.0001). Elderly individuals with normal cognitive function also exhibited varying degrees of improvement across the scales: the MMSE scale indicated a moderate improvement (MD = 1.60, 95% CI: 1.00–2.21, *p* < 0.0001), while the MoCA scale reflected the smallest effect (MD = 1.69, 95% CI: 0.39–2.00, *p* = 0.01) (see Figure 3 and Supplementary Figures S1A–D).

#### 3.4.2 Secondary outcomes

##### 3.4.2.1 Memory and attention function

Memory and attention functions were assessed using the Rey Auditory Verbal Learning Test (immediate recall, short-term delayed recall, long-term delayed recall) and the digit span test (digit span forward test, digit span backward test).

**Figure 4:**
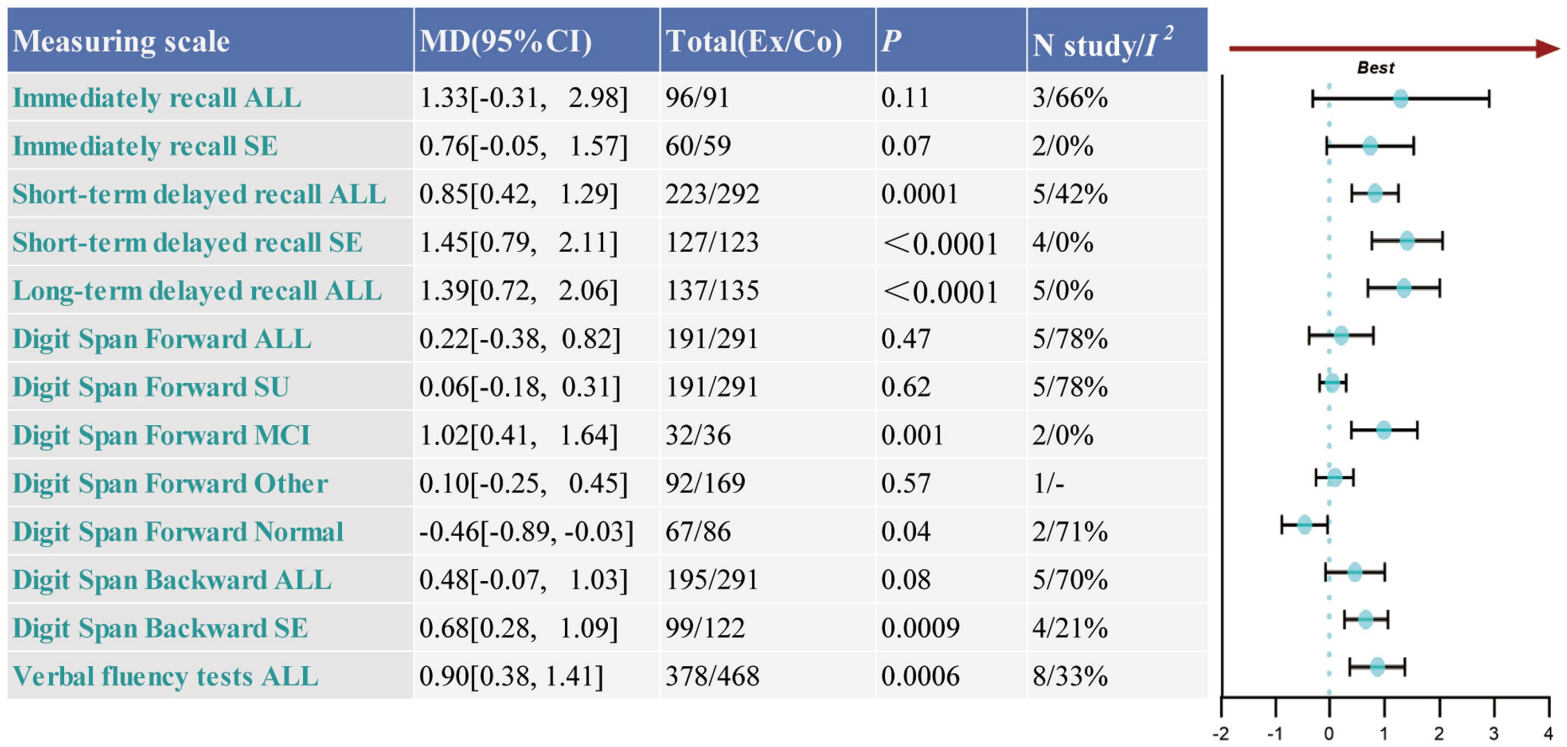
Collection of forest plots for memory and attention functions, as well as language functions. Ex, experimental group; Co, control group; All, meta-analysis results for all patients; SE, the results of sensitivity analysis; SU, results following sub-group analysis; MCI, mild cognitive impairment; HE, healthy elderly; Other, other types of cognitive impairments.

Three studies involving 187 participants assessed immediate recall, five studies with 515 participants evaluated short-term delayed recall, and five studies with 272 participants focused on long-term delayed recall to evaluate memory function. The analysis demonstrated that TCE interventions can enhance delayed recall function. Among the outcomes, long-term delayed recall showed the most substantial improvement (MD = 1.39, 95% CI: 0.72 to 2.06, *p* < 0.0001), followed by immediate recall (MD = 0.92, 95% CI: 0.12 to 1.72, *p* = 0.02) and short-term delayed recall (MD = 0.85, 95% CI: 0.42 to 1.29, *p* = 0.0001). The heterogeneity was relatively low for both short-term delayed recall (*p* = 0.14, *I*^2^ = 42%) and long-term delayed recall (*p* = 0.65, *I*^2^ = 0%), suggesting more reliable results. In contrast, the heterogeneity for immediate recall was higher (*p* = 0.05, *I*^2^ = 66%). Sensitivity analysis revealed that Xia (Xia et al., 2023) study significantly contributed to the heterogeneity. After excluding this study, the heterogeneity was markedly reduced (*p* = 0.34, *I*^2^ = 0%). Despite this, the analysis for immediate recall was not statistically significant (MD = 0.76, 95% CI: −0.05 to 1.57, *p* = 0.07) (see Figure 4 and Supplementary Figures S2A–E).

Five studies, involving a total of 482 participants, assessed the digit span forward test, while another five studies, with 486 participants, evaluated the digit span backward test. Both tests displayed considerable heterogeneity (Forward: *p* = 0.001, *I*^2^ = 78%; Backward: *p* = 0.009, *I*^2^ = 70%), necessitating the use of a random-effects model for the analysis. The findings indicated no significant improvement in performance on either test following the TCE intervention. Specifically, the digit span forward (MD = 0.06, 95% CI: −0.18 to 0.31, *p* = 0.62) and digit span backward (MD = 0.16, 95% CI: −0.53 to 0.39, *p* = 0.18) tests did not show any notable improvement in elderly individuals. Sensitivity analysis indicated that the study by Lam et al. (2014) had a significant influence on the heterogeneity of the backward digit span results. The exclusion of this study resulted in a reduction of heterogeneity (*p* = 0.28, *I*^2^ = 21%), and the findings remained statistically significant (MD = 0.68, 95% CI: 0.28 to 1.09, *p* = 0.0009). No significant differences were observed for the forward digit span, suggesting that the results were stable and robust. Further examination through a sub-group analysis of the forward digit span test revealed a positive effect in the MCI group (MD = 1.02, 95% CI: 0.41 to 1.64, *p* = 0.001). However, no significant improvements were detected in the healthy elderly group (MD = −0.46, 95% CI: −0.89 to −0.034, *p* = 0.004) or in the other cognitive dysfunction groups (MD = 0.10, 95% CI: −0.25 to 0.45, *p* = 0.57) (see Figure 4 and Supplementary Figures S2F–I).

##### 3.4.2.2 Language ability

This study analyzed eight publications, encompassing 846 participants, to compare the improvement in language ability between the two groups using categorical verbal fluency tests. The included studies demonstrated low heterogeneity (*p* = 0.16, *I*^2^ = 33%). The findings suggested that TCEs have a positive impact on enhancing language ability in older adults (MD: 0.90, 95% CI: 0.38 to 1.41) (refer to Figure 4 and Supplementary Figures S3A).

##### 3.4.2.3 Executive function

**Figure 5:**
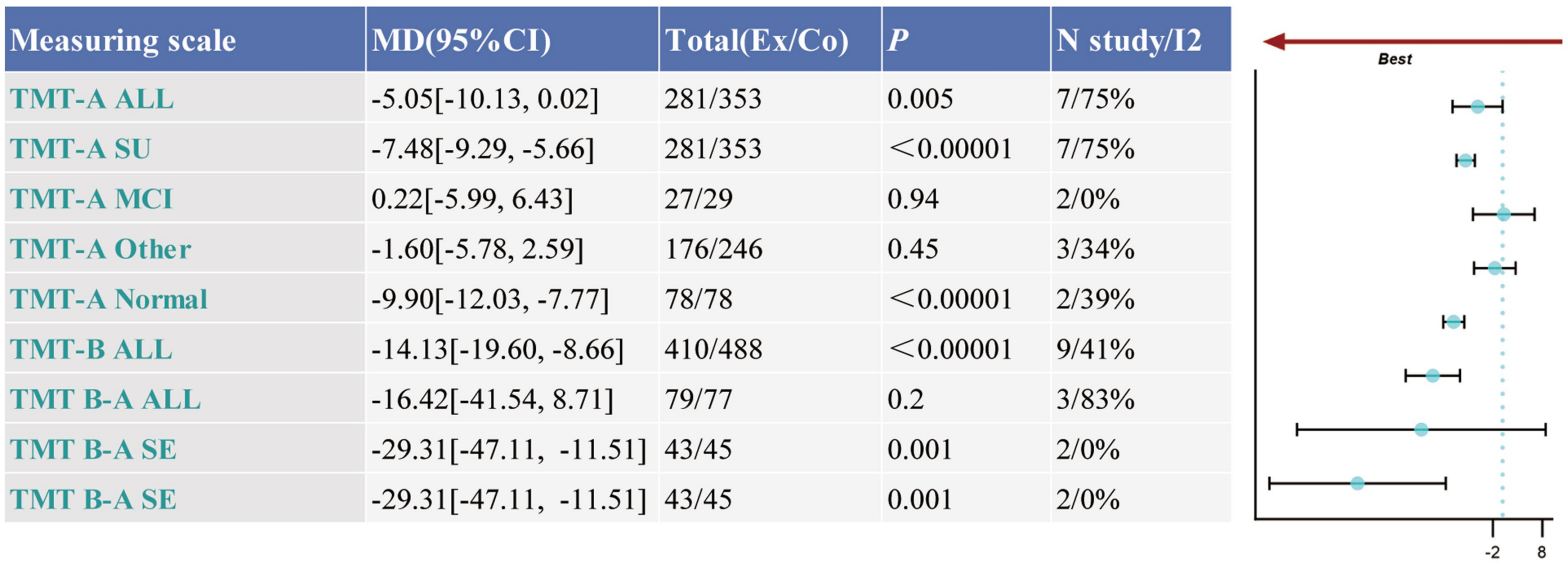
Forest plot collection for executive function. Ex, experimental group; Co, control group; All, meta-analysis results for all patients; SE, the results of sensitivity analysis; SU, results following sub-group analysis; MCI, mild cognitive impairment; HE, healthy elderly; Other, other types of cognitive impairments.

The Trail Making Test (TMT) was utilized to assess executive function. Seven studies, comprising 634 participants, evaluated the TMT-A component, while nine studies with 898 participants assessed the TMT-B component. Furthermore, three studies, involving 156 participants, examined the Part B-A difference score. The heterogeneity for TMT-A was substantial (*p* = 0.0006, *I*^2^ = 75%), and random-effects analysis revealed a significant improvement following intervention (MD = −7.48, 95% CI: −9.29 to −5.66, *p* < 0.00001), highlighting a marked effect on tasks measuring lower-level executive functions. Sensitivity analysis confirmed the robustness of these findings. Sub-group analysis revealed that a significant improvement in TMT-A performance was observed solely in the cognitively normal group (MD = −9.90, 95% CI: −12.03 to −7.77, *p* < 0.00001). TMT-B exhibited lower heterogeneity (*p* = 0.1, *I*^2^ = 41%), and fixed-effects analysis indicated a significant improvement in performance post-intervention (MD = −13.70, 95% CI: −16.06 to −11.35, *p* < 0.00001) for tasks assessing higher-level executive functions. The heterogeneity for the Part B-A difference score was higher (*p* = 0.003, *I*^2^ = 83%), and random-effects analysis showed no significant improvement following intervention (MD = 1.13, 95% CI: −0.61 to 2.87, *p* = 0.20), suggesting that the TMT B-A difference score did not show meaningful changes relative to simpler tasks. Sensitivity analysis identified that the study by Xia et al. (2023) had a substantial impact on heterogeneity. Upon its exclusion, the results demonstrated that TCEs significantly improved executive function (MD = −29.31, 95% CI: −47.11 to −11.51, *p* = 0.001) (refer to Figure 5 and Supplementary Figures S4A–E).

#### 3.4.3 Risk of bias

In the funnel plot analysis based on the MoCA outcome measure, the distribution of study points on either side of the mean effect size suggested that overall publication bias was likely minimal. However, the uneven distribution of a few data points, particularly at higher positive effect sizes, may have indicated a potential presence of some publication bias (see Supplementary Figure S5).

## 4 Discussion

This study included 29 randomized controlled trials that systematically assessed the effects of TCEs on overall cognitive function and specific cognitive domains in older adults. The results indicated that TCEs were superior to the control group in enhancing overall cognitive function, delayed recall, executive function, and language abilities in older adults. Sub-group analysis further revealed that the effectiveness of TCEs varied among older adults depending on their cognitive status. No adverse events were reported in any of the included studies, suggesting that TCEs are a safe intervention for cognitive function. This finding is consistent with the conclusions of studies by Park et al. (2023) and Zhang et al. (2018).

The findings of this study indicate a modest improvement in overall cognitive function following TCE intervention in older adults, in contrast to the more substantial enhancements observed in the study by Cai et al. (2023). This discrepancy may be attributed to differences in the sample populations. While our study included cognitively healthy elderly participants, the study by Cai et al. (2023) was restricted to individuals with cognitive impairments. Cognitively normal older adults generally exhibit higher baseline cognitive function, with more stable neural networks and brain activity, leaving limited scope for further improvement (Lenze et al., 2022). Consequently, the effect of TCE intervention appears to be confined to maintaining or slightly enhancing existing cognitive functions, without inducing substantial changes to their overall cognitive state. Conversely, individuals with cognitive impairments typically present with lower baseline cognitive abilities, and their brain networks often demonstrate reduced efficiency and connectivity (Wang S. et al., 2024). TCEs, which require participants to engage in tasks demanding sustained attention and fine motor coordination, activate key brain regions such as the prefrontal cortex and hippocampus. This activation enhances neural plasticity, strengthens network connectivity, and promotes synchrony between brain regions, thereby unlocking the brain’s potential and leading to more pronounced cognitive improvements (Cai et al., 2023; Zhang et al., 2023). Sub-group analyses within this study further corroborate this hypothesis, as elderly individuals with cognitive impairment exhibited more significant cognitive gains following TCE intervention than their cognitively healthy counterparts.

Previous studies have suggested that a 12-week TCE intervention can enhance memory function in older adults (Tao et al., 2016). This study further validated this finding using the auditory verbal learning test and digit span tests. Our sub-group analysis also uncovered an intriguing phenomenon: the improvement in memory function appeared to be influenced by the cognitive status of the participant. For patients with MCI, significant gains were observed in both immediate and delayed recall, as well as in the forward digit span, indicating that TCE interventions are particularly beneficial for those with more pronounced cognitive impairments. Other groups with cognitive impairments, such as those with subjective cognitive decline and amnestic cognitive impairment, also showed notable improvements in immediate and delayed recall, further supporting the potential of TCEs in facilitating memory recovery. By contrast, healthy older adults exhibited significant progress only in delayed recall, which may be attributable to their higher baseline memory levels. Despite the positive effects of TCEs across several dimensions of memory, this study did not observe significant improvements in the backward digit span test. This outcome contrasts with findings from a retrospective study on long-term TCE practice, which demonstrated that after more than 3 years of TCE practice, the backward digit span scores of the experimental group were significantly higher than those of the control group (Jor’dan et al., 2018). This discrepancy may be due to the shorter intervention duration in our study, which ranged from only three to 6 months, potentially limiting the accumulation of significant long-term effects. Consequently, extending the intervention period should be considered to further investigate the impact of TCEs on complex memory functions.

Executive function is intimately connected with lower-level cognitive functions, such as perception, memory, and attention. While executive function governs these lower-level processes, thus facilitating goal-directed behavior, the optimization of these cognitive functions, in turn, enhances the efficiency and flexibility of executive function (Friedman and Miyake, 2017; Harvey, 2019). TCEs require practitioners to continually shift their attention to maintain the coherence of movement sequences and to synchronize breathing with the rhythm of movements (Lan et al., 2023; Yang et al., 2020). This process activates and optimizes lower-level cognitive functions, such as the flexible allocation of attention, immediate memory recall, and heightened bodily awareness, thereby enhancing overall cognitive abilities (Friedman and Miyake, 2017; Ren et al., 2021). The findings of this study corroborate this perspective, as older adults who participated in TCE interventions performed significantly better than the control group in both the TMT B and TMT B-A tests, indicating that TCEs can substantially improve executive function in older adults.

This study found that TCEs significantly improved language function in older adults, consistent with the findings of Wu et al. (2019). Although this study did not directly investigate the specific relationship between language and other cognitive functions, the existing literature suggests that language is closely intertwined with executive and memory functions (Banjac et al., 2021; Friedman and Sterling, 2019). These cognitive processes are pivotal in the generation, comprehension, and organization of language, collaboratively ensuring the smooth and accurate transmission of information, which is crucial for language fluency (Perrone-Bertolotti et al., 2017; Pu et al., 2020). Given the comprehensive cognitive benefits of TCEs, we speculate that the observed improvements in language function may be partially attributed to its positive effects on executive and memory functions.

While this study utilized a rigorous selection of randomized controlled trials to ensure the validity of causal relationships and the quality of evidence, several limitations should be acknowledged. As TCEs are, by definition, Chinese exercises, the study population was predominantly Chinese, which may limit the generalizability of the findings to the global elderly population. Moreover, fully implementing a double-blind design in exercise intervention studies is inherently challenging, potentially introducing bias that could undermine the robustness of the conclusions. Furthermore, the diverse forms of TCEs, along with variations in the duration, intensity, and frequency of interventions, may have influenced the consistency of the study results.

Future experimental research should consider the inclusion of more diverse populations, including those from different cultural backgrounds, and explore ways to standardize exercise protocols (e.g., duration, intensity, and frequency) to enhance the generalizability and reproducibility of findings. Additionally, the adoption of more rigorous trial designs, such as multicenter trials and double-blind interventions, could help mitigate potential biases and improve the robustness of the evidence base.

## 5 Conclusion

This study demonstrated that TCEs significantly improve cognitive function in older adults, particularly those with MCI. The varied effects of TCEs across different cognitive domains suggest that individual differences in cognitive status should be carefully considered when designing intervention programs in order to achieve precise and personalized outcomes.

## Data Availability

The original contributions presented in the study are included in the article/Supplementary material, further inquiries can be directed to the corresponding author.
