# Supplementary material for: A meta-analysis of the influence of traditional Chinese exercises on cognitive function in the elderly
Source: Front Psychol. 2025 Apr 8;16:1516197. doi: 10.3389/fpsyg.2025.1516197 (PMC12012620; doi:10.3389/fpsyg.2025.1516197)
Supplement: Supplementary file 1 [file Data_Sheet_1.docx]

Supplementary Material

# Supplementary Figures

**Supplementary Figure S1.** Overall cognitive function

# (A) Forest map of MOCA scale


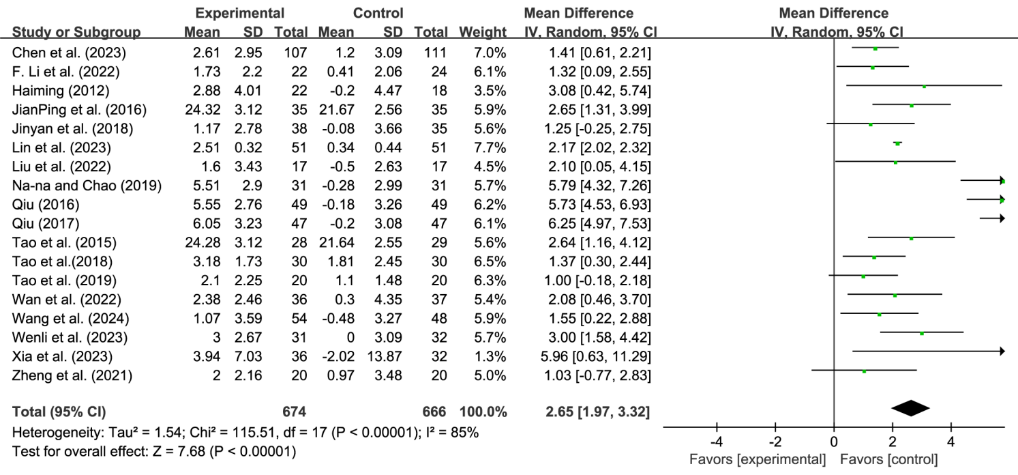


# Subgroup analysis forest map of MOCA scale


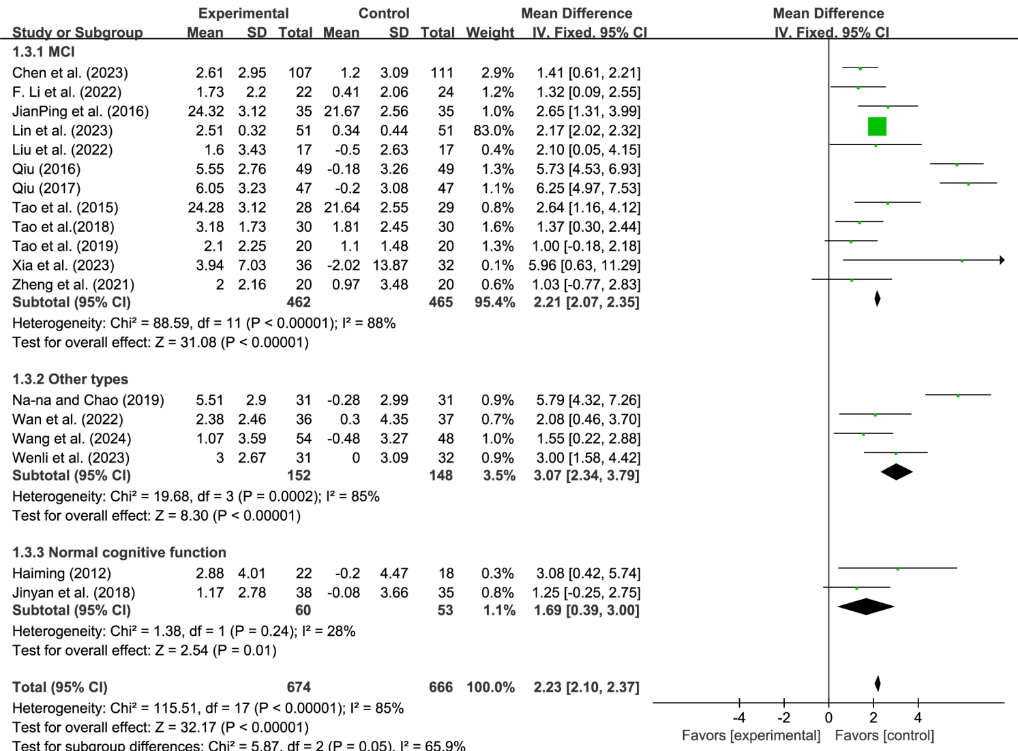


1. Forest map of MMSE scale

**
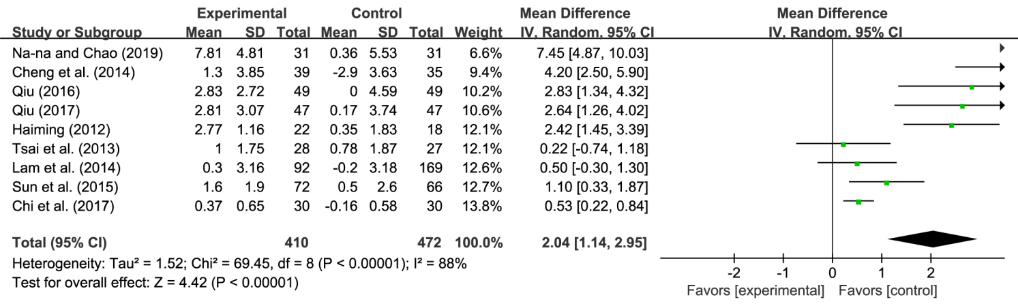
**

1. Subgroup analysis forest map of MMSE scale

**
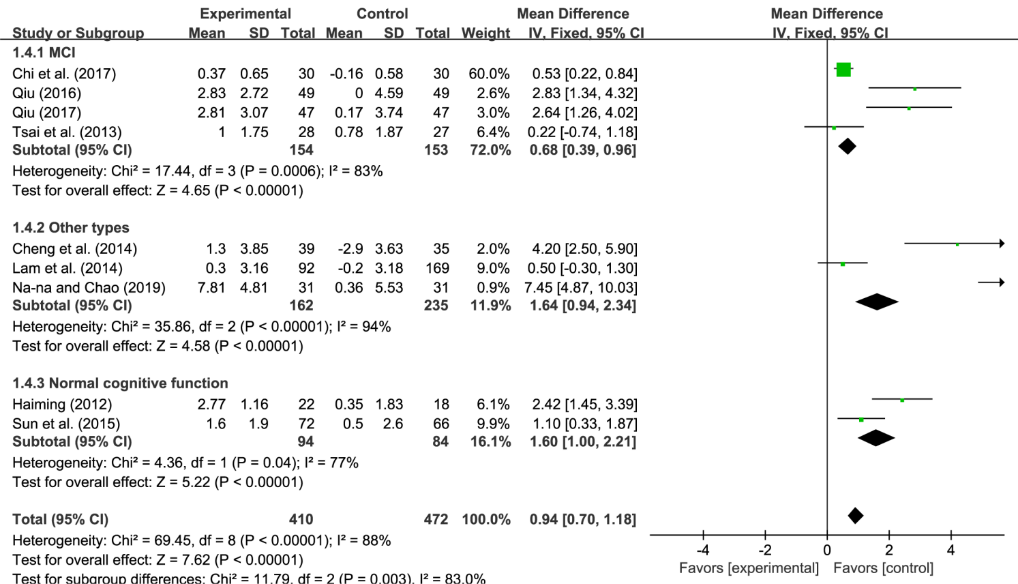
**

**Supplementary Figure S2.** Memory and attention function

1. Forest map of immediately recall test


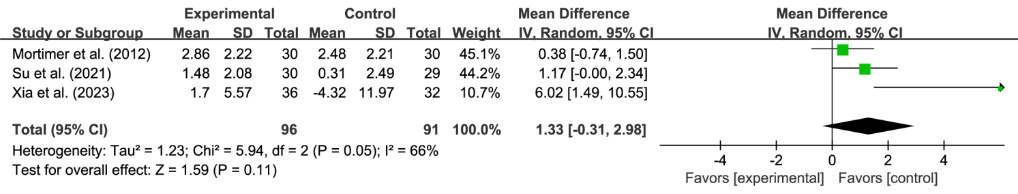


1. Forest map after sensitivity analysis of immediately recall test


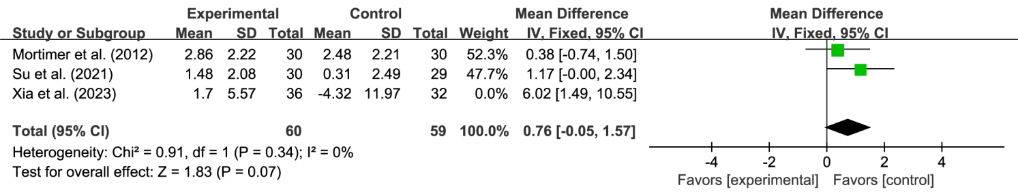


1. Forest map of short-term delayed recall test


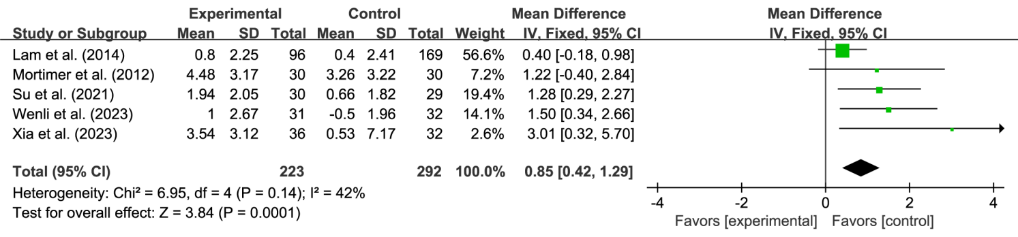


1. Forest map after sensitivity analysis of short-term delayed recall test


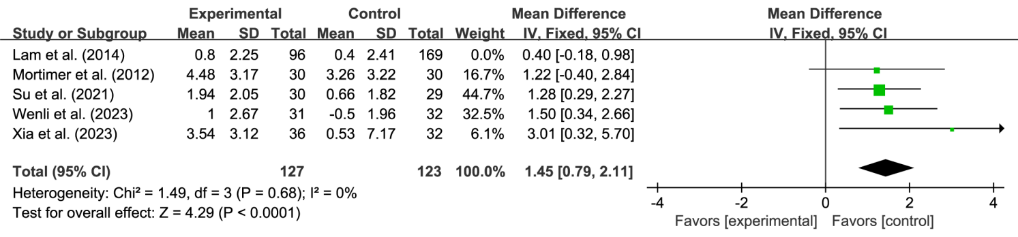


1. Forest map of long-term delayed recall test


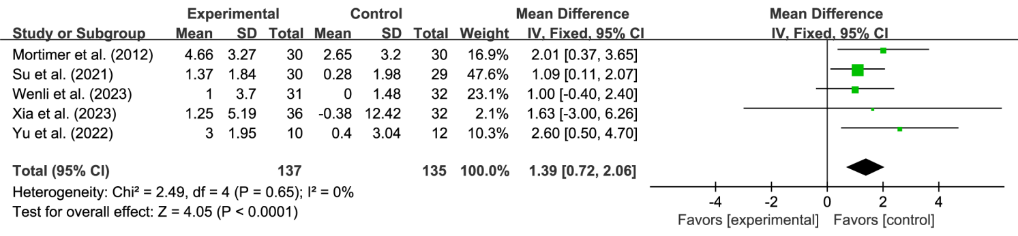


1. Forest map of digit span forward test


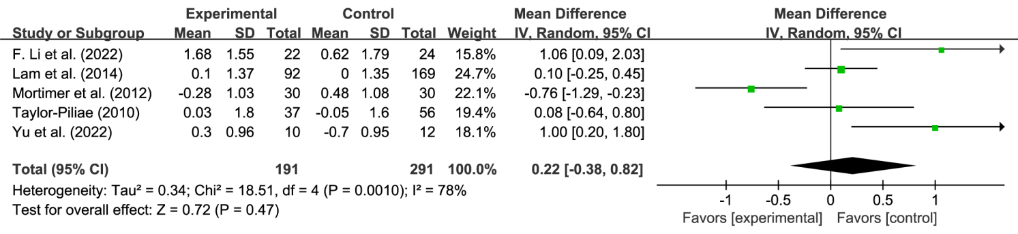


1. Subgroup analysis forest map of digit span forward test


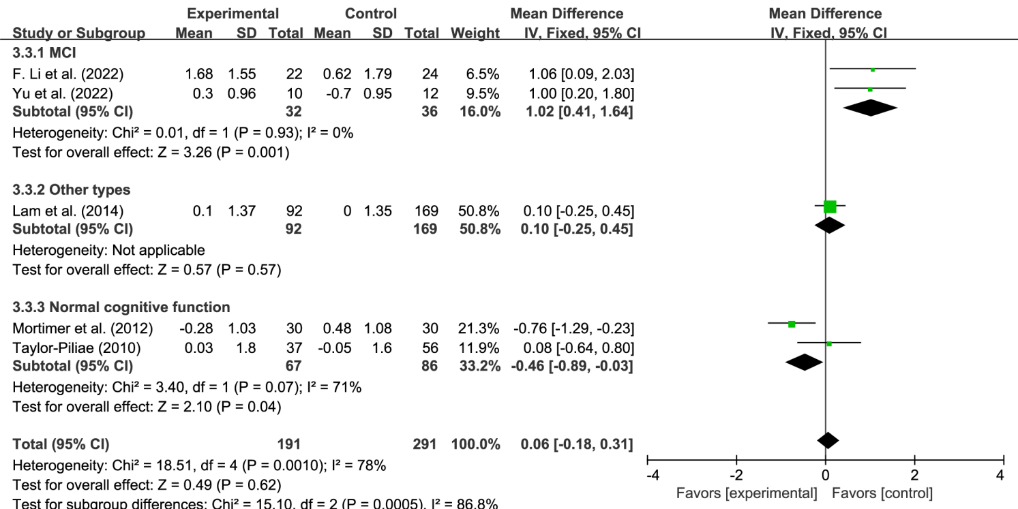


1. Forest map of digit span backward test


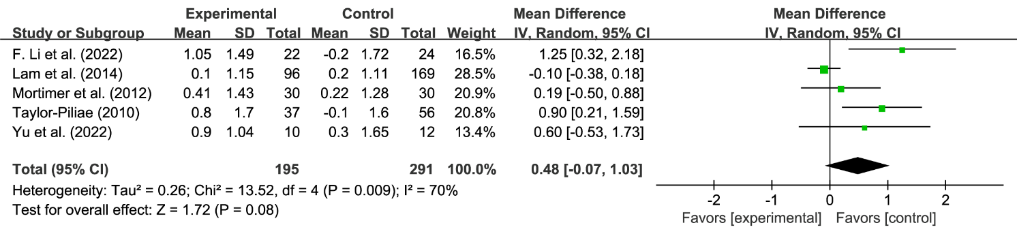


1. Forest map after sensitivity analysis of digit span backward test


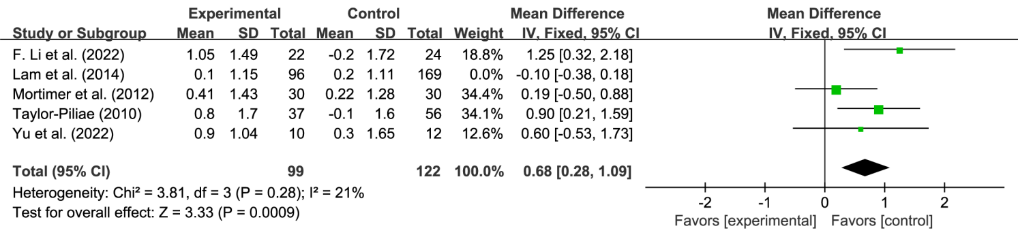


**Supplementary Figure S3.** Language ability

1. Forest map of categorical verbal fluency tests


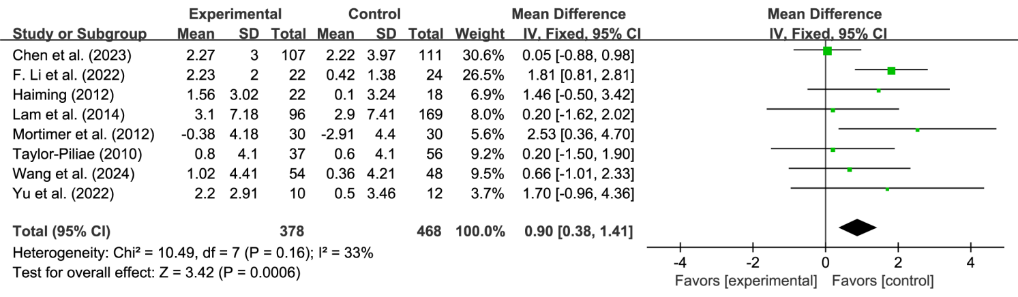


#### Supplementary Figure S4. Executive function

1. Forest map of TMT-A


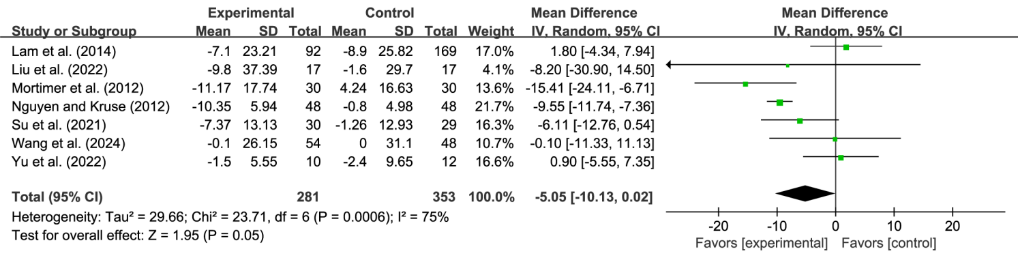


(B）Subgroup analysis forest map of TMT-A


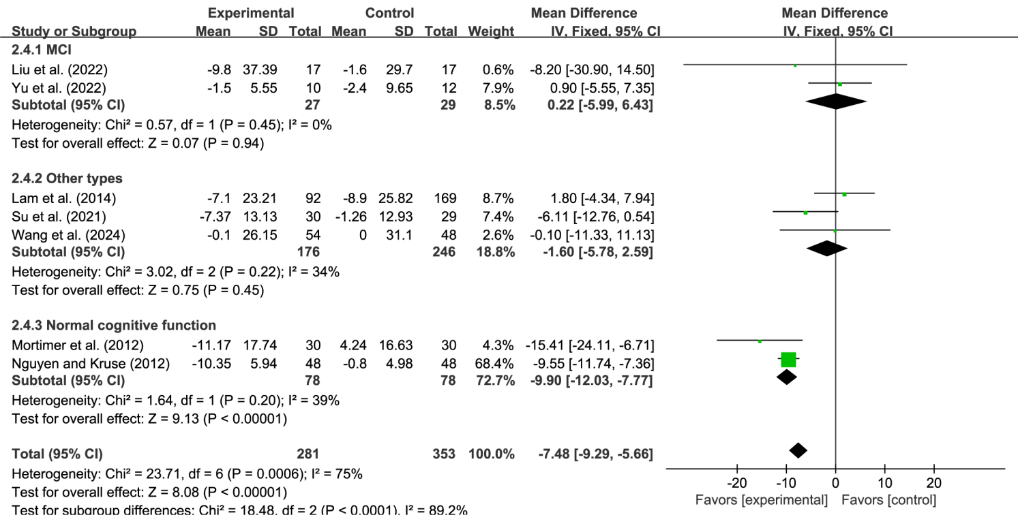


1. Forest map of TMT-B


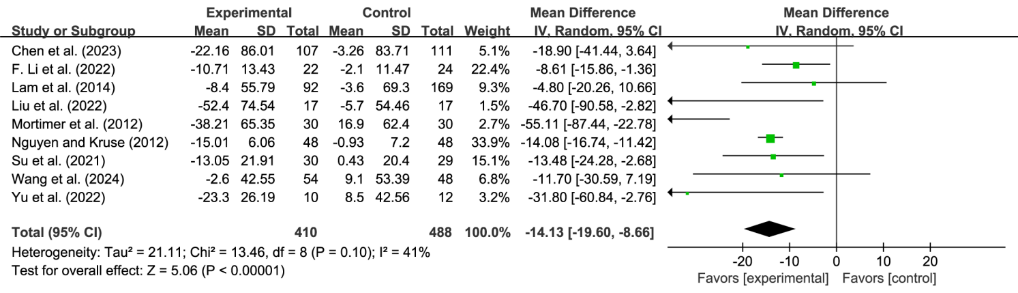


1. Forest map of TMT B-A


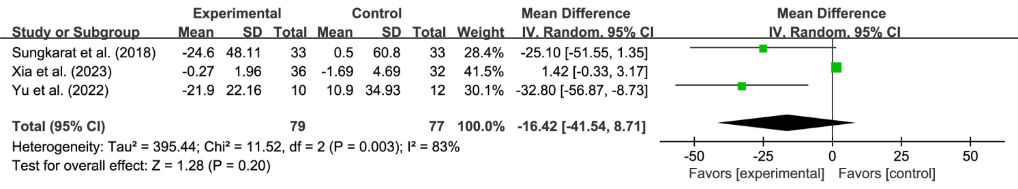


1. Forest map after sensitivity analysis of TMT B-A


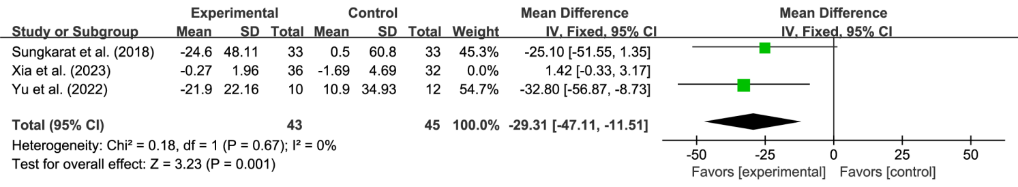


**Supplementary Figure S5.** Funnel plot to detect the publication bias of included studies


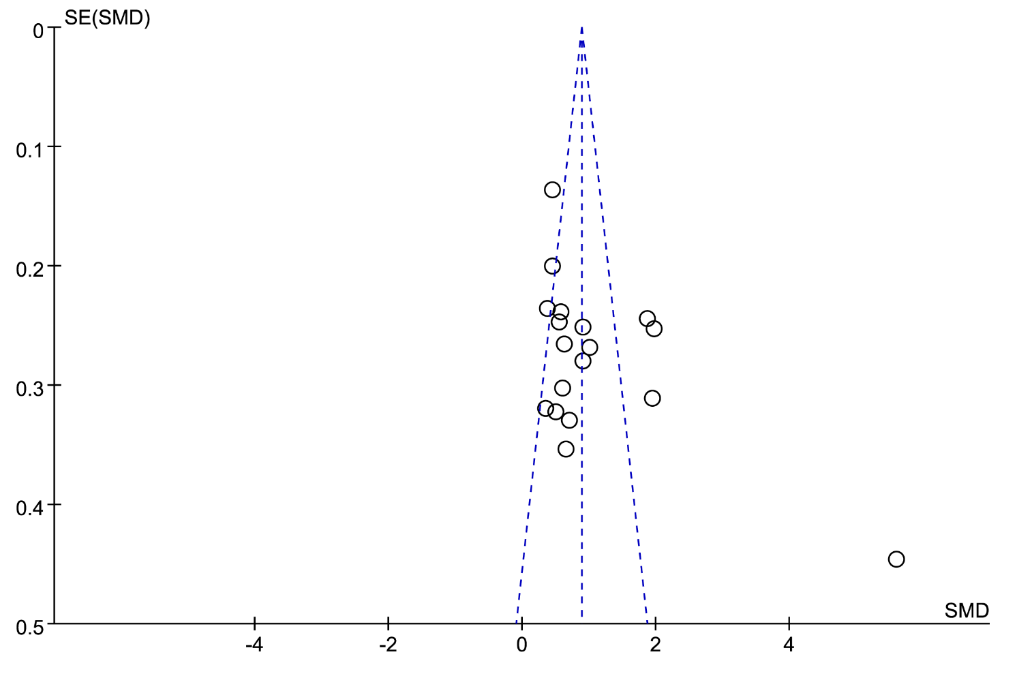


# Supplementary Tables

**Table S1.** PRISMA NMA Checklist of Items to Include When Reporting a Systematic Review Involving a Network Meta-analysis


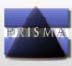
**PRISMA 2020 Checklist**

| **Section and Topic** | **Item #** | **Checklist item** | **Location where item is reported** |
| --- | --- | --- | --- |
| **TITLE** | | |  |
| Title | 1 | Identify the report as a systematic review. | 1 |
| **ABSTRACT** | | |  |
| Abstract | 2 | See the PRISMA 2020 for Abstracts checklist. | 1 |
| **INTRODUCTION** | | |  |
| Rationale | 3 | Describe the rationale for the review in the context of existing knowledge. | 2-3 |
| Objectives | 4 | Provide an explicit statement of the objective(s) or question(s) the review addresses. | 3 |
| **METHODS** | | |  |
| Eligibility criteria | 5 | Specify the inclusion and exclusion criteria for the review and how studies were grouped for the syntheses. | 4 |
| Information sources | 6 | Specify all databases, registers, websites, organisations, reference lists and other sources searched or consulted to identify studies. Specify the date when each source was last searched or consulted. | 3 |
| Search strategy | 7 | Present the full search strategies for all databases, registers and websites, including any filters and limits used. | Supplementary Table S1 |
| Selection process | 8 | Specify the methods used to decide whether a study met the inclusion criteria of the review, including how many reviewers screened each record and each report retrieved, whether they worked independently, and if applicable, details of automation tools used in the process. | 4 |
| Data collection process | 9 | Specify the methods used to collect data from reports, including how many reviewers collected data from each report, whether they worked independently, any processes for obtaining or confirming data from study investigators, and if applicable, details of automation tools used in the process. | 4 |
| Data items | 10a | List and define all outcomes for which data were sought. Specify whether all results that were compatible with each outcome domain in each study were sought (e.g. for all measures, time points, analyses), and if not, the methods used to decide which results to collect. | 19-24 |
|  | 10b | List and define all other variables for which data were sought (e.g. participant and intervention characteristics, funding sources). Describe any assumptions made about any missing or unclear information. | 19-24 |
| Study risk of bias assessment | 11 | Specify the methods used to assess risk of bias in the included studies, including details of the tool(s) used, how many reviewers assessed each study and whether they worked independently, and if applicable, details of automation tools used in the process. | 6 |
| Effect measures | 12 | Specify for each outcome the effect measure(s) (e.g. risk ratio, mean difference) used in the synthesis or presentation of results. | Supplementary Figure 2 |
| Synthesis methods | 13a | Describe the processes used to decide which studies were eligible for each synthesis (e.g. tabulating the study intervention characteristics and comparing against the planned groups for each synthesis (item #5)). | 5 |
|  | 13b | Describe any methods required to prepare the data for presentation or synthesis, such as handling of missing summary statistics, or data conversions. | 5 |
|  | 13c | Describe any methods used to tabulate or visually display results of individual studies and syntheses. | 5 |
|  | 13d | Describe any methods used to synthesize results and provide a rationale for the choice(s). If meta-analysis was performed, describe the model(s), method(s) to identify the presence and extent of statistical heterogeneity, and software package(s) used. | 5 |
|  | 13e | Describe any methods used to explore possible causes of heterogeneity among study results (e.g. subgroup analysis, meta-regression). | 5 |
|  | 13f | Describe any sensitivity analyses conducted to assess robustness of the synthesized results. | 5 |
| Reporting bias assessment | 14 | Describe any methods used to assess risk of bias due to missing results in a synthesis (arising from reporting biases). | 5 |
| Certainty assessment | 15 | Describe any methods used to assess certainty (or confidence) in the body of evidence for an outcome. | 5 |
| **RESULTS** | | |  |
| Study selection | 16a | Describe the results of the search and selection process, from the number of records identified in the search to the number of studies included in the review, ideally using a flow diagram. | 3 |
|  | 16b | Cite studies that might appear to meet the inclusion criteria, but which were excluded, and explain why they were excluded. | No |
| Study characteristics | 17 | Cite each included study and present its characteristics. | 19–24 |
| Risk of bias in studies | 18 | Present assessments of risk of bias for each included study. | 6 |
| Results of individual studies | 19 | For all outcomes, present, for each study: (a) summary statistics for each group (where appropriate) and (b) an effect estimate and its precision (e.g. confidence/credible interval), ideally using structured tables or plots. | 8 |
| Results of syntheses | 20a | For each synthesis, briefly summarise the characteristics and risk of bias among contributing studies. | Supplementary Figure S6 |
|  | 20b | Present results of all statistical syntheses conducted. If meta-analysis was done, present for each the summary estimate and its precision (e.g. confidence/credible interval) and measures of statistical heterogeneity. If comparing groups, describe the direction of the effect. | No |
|  | 20c | Present results of all investigations of possible causes of heterogeneity among study results. | No |
|  | 20d | Present results of all sensitivity analyses conducted to assess the robustness of the synthesized results. | No |
| Reporting biases | 21 | Present assessments of risk of bias due to missing results (arising from reporting biases) for each synthesis assessed. | Supplementary Figure 2 |
| Certainty of evidence | 22 | Present assessments of certainty (or confidence) in the body of evidence for each outcome assessed. | Supplementary Figure 2 |
| **DISCUSSION** | | |  |
| Discussion | 23a | Provide a general interpretation of the results in the context of other evidence. | 8–10 |
|  | 23b | Discuss any limitations of the evidence included in the review. | 10 |
|  | 23c | Discuss any limitations of the review processes used. | NO |
|  | 23d | Discuss implications of the results for practice, policy, and future research. | 8 |
| **OTHER INFORMATION** | | |  |
| Registration and protocol | 24a | Provide registration information for the review, including register name and registration number, or state that the review was not registered. | 3 |
|  | 24b | Indicate where the review protocol can be accessed, or state that a protocol was not prepared. | no |
|  | 24c | Describe and explain any amendments to information provided at registration or in the protocol. | no |
| Support | 25 | Describe sources of financial or non-financial support for the review, and the role of the funders or sponsors in the review. | 11 |
| Competing interests | 26 | Declare any competing interests of review authors. | 11 |
| Availability of data, code and other materials | 27 | Report which of the following are publicly available and where they can be found: template data collection forms; data extracted from included studies; data used for all analyses; analytic code; any other materials used in the review. | 11 |

*From:*  Page MJ, McKenzie JE, Bossuyt PM, Boutron I, Hoffmann TC, Mulrow CD, et al. The PRISMA 2020 statement: an updated guideline for reporting systematic reviews. BMJ 2021;372:n71. doi: 10.1136/bmj.n71

**Table S2.** Literature Search Strategy

| **Pubmed** | Search: (((((((((((((((((((((((((((((((((((((((((((((((((((((((((((Cognition[MeSH Terms]) OR (Cognition)) OR (Cognitions)) OR (Cognitive Function)) OR (Cognitive Functions)) OR (Function, Cognitive)) OR (Functions, Cognitive)) OR (Memory[MeSH Terms])) OR (Memory)) OR (Recall)) OR (Learning[MeSH Terms])) OR (Learning)) OR (Phenomenography)) OR (Executive Function[MeSH Terms])) OR (Executive Function)) OR (Executive Functions)) OR (Function, Executive)) OR (Functions, Executive)) OR (Executive Control)) OR (Executive Controls)) OR (Problem Solving[MeSH Terms])) OR (Problem Solving)) OR (Perception[MeSH Terms])) OR (Perception)) OR (Sensory Processing)) OR (Processing, Sensory)) OR (Perceptions)) OR (Attention[MeSH Terms])) OR (Attention)) OR (Focus of Attention)) OR (Attention Focus)) OR (Selective Attention)) OR (Attention, Selective)) OR (Selective Attentions)) OR (Social Attention)) OR (Attention, Social)) OR (Concentration)) OR (Concentrations)) OR (Awareness[MeSH Terms])) OR (Awareness)) OR (Awarenesses)) OR (Situational Awareness)) OR (Awareness, Situational)) OR (Awarenesses,Situational)) OR (Situational Awarenesses)) OR (Situation Awareness)) OR (Awareness, Situation)) OR (Awarenesses,Situation)) OR (Situation Awarenesses)) OR (comprehension[MeSH Terms])) OR (comprehension)) OR (Understanding)) OR (Readability)) OR (Language[MeSH Terms])) OR (Language)) OR (Languages)) OR (Dialect)) OR (Dialects)) AND ((((((((((((((((((((Qigong[MeSH Terms]) OR (Qigong)) OR (Qi Gong)) OR (Ch'i Kung)) OR (Tai Chi[MeSH Terms])) OR (Tai Chi)) OR (Tai-ji)) OR (Tai Ji)) OR (Chi, Tai)) OR (Tai Ji Quan)) OR (Ji Quan, Tai)) OR (Quan, Tai Ji)) OR (Taiji)) OR (Taijiquan)) OR (T'ai Chi)) OR (Tai Chi Chuan)) OR (Baduanjin)) OR (Yijinjing)) OR (Wuqinxi)) OR (Liuzijue))) AND (((((((Randomized Controlled Trial[MeSH Terms]) OR (Randomized Controlled Trial)) OR (random allocation)) OR (random*)) OR (Allocation, Random)) OR (Control*)) OR (Comparison)) |
| --- | --- |
| **Web of Science** | 1: TS=(Cognition OR Cognitions OR Cognitive Function OR Cognitive Functions OR Function, Cognitive OR Functions, Cognitive OR Memory OR Recall OR Learning OR Phenomenography OR Executive Function OR Executive Functions OR Function, Executive OR Functions, Executive OR Executive Control OR Executive Controls OR Problem Solving OR Perception OR Sensory Processing OR Processing, Sensory OR Perceptions OR Attention OR Focus of Attention OR Attention Focus OR Selective Attention OR Attention, Selective OR Selective Attentions OR Social Attention OR Attention, Social OR Concentration OR Concentrations OR Awareness OR Awarenesses OR Situational Awareness OR Awareness, Situational OR Awarenesses,Situational OR Situational Awarenesses OR Situation Awareness OR Awareness, Situation OR Awarenesses,Situation OR Situation Awarenesses OR comprehension OR Understanding OR Readability OR Language OR Languages OR Dialect OR Dialects ) and Preprint Citation Index (Exclude – Database) Results: 29474349  2: TS=( Qigong OR Qigong OR Ch'i Kung OR Tai Chi OR Tai-ji OR Tai Ji OR Chi, Tai OR Tai Ji Quan OR Ji Quan, Tai OR Quan, Tai Ji OR Taiji OR Taijiquan OR T'ai Chi OR Tai Chi Chuan OR Baduanjin OR Yijinjing OR Wuqinxi OR Liuzijue ) and Preprint Citation Index (Exclude – Database) Results: 9869  3: TS=( Comparison OR Trial OR Randomized Controlled Trial OR random allocation OR random* OR Allocation, Random ) and Preprint Citation Index (Exclude – Database) Results: 8826349  4: #1 AND #2 AND #3 and Preprint Citation Index (Exclude – Database) Results: 1168 |
| **Cochrane** | #1 MeSH descriptor: [Qigong] explode all trees 155  #2 (Qigong):ti,ab,kw OR (Ch'i Kung):ti,ab,kw OR (Qi Gong):ti,ab,kw 810  #3 MeSH descriptor: [Tai Ji] explode all trees 582  #4 (Tai Ji):ti,ab,kw OR (Tai Chi Chuan):ti,ab,kw OR (Ji Quan, Tai):ti,ab,kw OR (Tai Ji Quan):ti,ab,kw OR (Quan, Tai Ji):ti,ab,kw 727  #5 (T'ai Chi):ti,ab,kw OR (Chi, Tai):ti,ab,kw OR (Tai-ji):ti,ab,kw OR (Tai Chi):ti,ab,kw OR (Taiji):ti,ab,kw 1917  #6 (badaunjin):ti,ab,kw OR (yijinjing):ti,ab,kw OR (wuqinxi):ti,ab,kw OR (liuzijue):ti,ab,kw 150  #7 #1 OR #2 OR #3 OR #4 OR #5 OR #6 2685  #8 MeSH descriptor: [Cognition] explode all trees 16241  #9 (Cognition):ti,ab,kw OR (Cognitions):ti,ab,kw OR (Function, Cognitive):ti,ab,kw OR (Functions, Cognitive):ti,ab,kw OR (Cognitive Function):ti,ab,kw 55454  #10 (Cognitive Functions):ti,ab,kw 7835  #11 MeSH descriptor: [Memory] explode all trees 9947  #12 (Memory):ti,ab,kw OR (Recall):ti,ab,kw 39335  #13 MeSH descriptor: [Learning] explode all trees 22799  #14 (Learning):ti,ab,kw OR (Phenomenography):ti,ab,kw 38101  #15 MeSH descriptor: [Executive Function] explode all trees 1841  #16 (Executive Function):ti,ab,kw OR (Function, Executive):ti,ab,kw OR (Executive Control):ti,ab,kw OR (Executive Controls):ti,ab,kw OR (Executive Functions):ti,ab,kw 9794  #17 (Functions, Executive):ti,ab,kw 3096  #18 MeSH descriptor: [Problem Solving] explode all trees 2029  #19 (Problem Solving):ti,ab,kw 7090  #20 MeSH descriptor: [Perception] explode all trees 24033  #21 (Perception):ti,ab,kw OR (Sensory Processing):ti,ab,kw OR (Processing, Sensory):ti,ab,kw OR (Perceptions):ti,ab,kw 41399  #22 MeSH descriptor: [Attention] explode all trees 6988  #23 (Attention):ti,ab,kw OR (Attention, Social):ti,ab,kw OR (Social Attention):ti,ab,kw OR (Attention Focus):ti,ab,kw OR (Focus of Attention):ti,ab,kw 41343  #24 (Selective Attentions):ti,ab,kw OR (Selective Attention):ti,ab,kw OR (Attention, Selective):ti,ab,kw 1932  #25 MeSH descriptor: [Awareness] explode all trees 1234  #26 (Awareness):ti,ab,kw OR (Situation Awareness):ti,ab,kw OR (Awarenesses):ti,ab,kw OR (Awareness, Situational):ti,ab,kw OR (Awareness, Situation):ti,ab,kw 15242  #27 (Situational Awarenesses):ti,ab,kw OR (Awarenesses, Situation):ti,ab,kw OR (Awarenesses, Situational):ti,ab,kw OR (Situational Awareness):ti,ab,kw OR (Situation Awarenesses):ti,ab,kw 142  #28 MeSH descriptor: [Comprehension] explode all trees 939  #29 (Comprehension):ti,ab,kw OR (Readability):ti,ab,kw OR (Understanding):ti,ab,kw 32535  #30 MeSH descriptor: [Language] explode all trees 7688  #31 (Language):ti,ab,kw OR (Languages):ti,ab,kw OR (Dialect):ti,ab,kw OR (Dialects):ti,ab,kw 26127  #32 #8 OR #9 OR #10 OR #11 OR #12 OR #13 OR #14 OR #15 OR #16 OR #17 OR #18 OR #19 OR #20 OR #21 OR #22 OR #23 OR #24 OR #25 OR #26 OR #27 OR #28 OR #29 OR #30 OR #31 240262  #33 MeSH descriptor: [Random Allocation] explode all trees 26023  #34 (Random Allocation):ti,ab,kw OR (Allocation, Random):ti,ab,kw OR (Randomization):ti,ab,kw OR (random*):ti,ab,kw 1337387  #35 #33 OR #34 1337387  #36 #7 AND #32 AND #35 532 |
| **Embase** | #4 #1 AND #2 AND #3 745  #3  'randomized controlled trial'/exp OR 'controlled trial, randomized' OR 'randomised controlled study' OR 'randomised controlled trial' OR 'randomized controlled study' OR 'trial, randomized controlled' OR 'randomized controlled trial' 1124876  #2  'cognition'/exp OR 'cognitive accessibility' OR 'cognitive balance' OR 'cognitive dissonance' OR 'cognitive function' OR 'cognitive structure' OR 'cognitive symptoms' OR 'cognitive task' OR 'cognitive thinking' OR 'neurobehavioural manifestations' OR 'volition' OR 'cognition' OR 'memory'/exp OR 'item recall' OR 'memory function' OR 'nonspatial memory' OR 'remembering' OR 'reminiscence' OR 'memory' OR 'learning'/exp OR 'absolute learning' OR 'child learning' OR 'knowledge acquisition' OR 'learning situation' OR 'relearning' OR 'learning' OR 'executive function'/exp OR 'cognitive control' OR 'executive control' OR 'executive function' OR 'problem solving'/exp OR 'problem solving' OR 'perception'/exp OR 'perception size' OR 'perceptual performance' OR 'perceptual processing' OR 'perceptual task' OR 'size detection' OR 'size discrimination' OR 'size perception' OR 'social perception' OR 'weight perception' OR 'perception' OR 'attention'/exp OR 'attentiveness' OR 'attention' OR 'awareness'/exp OR 'awareness' OR 'comprehension'/exp OR 'passive comprehension' OR 'understanding' OR 'comprehension' OR 'language'/exp OR 'figurative language' OR 'language analysis' OR 'language arts' OR 'language group' OR 'language' 5896733  #1 'tai chi'/exp OR 'tai chi chuan' OR 'tai ji' OR 'taiji quan' OR 'taijiquan' OR 'tai chi' OR 'qigong'/exp OR 'chi kung' OR 'chigung' OR 'qi gong' OR 'qigong' 6696 |
| **EBSCO** | #1 SU ( Cognition OR memory OR learning OR executive function OR problem solving OR perception OR attention OR awareness OR comprehension OR language ) OR TX ( cognitive accessibility OR cognitive balance OR cognitive dissonance OR cognitive function OR cognitive structure OR cognitive symptoms OR cognitive task OR cognitive thinking OR neurobehavioural manifestations OR volition OR cognition OR item recall OR memory function OR nonspatial memory OR remembering OR reminiscence OR memory OR absolute learning OR child learning OR knowledge acquisition OR learning situation OR relearning OR learning OR cognitive control OR executive control OR executive function OR problem solving OR perception size OR perceptual performance OR perceptual processing OR perceptual task OR size detection OR size discrimination OR size perception OR social perception OR weight perception OR perception OR attentiveness OR attention OR awareness OR passive comprehension OR understanding OR comprehension OR figurative language OR language analysis OR language arts OR language group OR language ) 531259  #2 SU ( tai chi OR qigong ) OR TX ( tai chi chuan OR tai ji OR taiji quan OR taijiquan OR tai chi OR chi kung OR chigung OR qi gong OR qigong ) 5548  #3 SU randomized controlled OR TX ( controlled trial, randomized OR randomised controlled study OR randomised controlled trial OR randomized controlled study OR trial, randomized controlled OR randomized controlled trial ) 61022  #4 S1 AND S2 AND S3 946 |
